# The multiple roles of autophagy in uveal melanoma and the microenvironment

**DOI:** 10.1007/s00432-023-05576-3

**Published:** 2024-03-11

**Authors:** Bo Liu, Xueting Yao, Yu Shang, Jinhui Dai

**Affiliations:** 1 Department of Ophthalmology, Zhongshan Hospital Affiliated to Fudan University, Shanghai, China; 2 Department of Laboratory Medicine, Longhua Hospital, Shanghai University of Traditional Chinese Medicine, Shanghai, China

**Keywords:** Autophagy, Uveal melanoma, Microenvironment, Drug target

## Abstract

**Purpose:**

Uveal melanoma (UM) is the most common primary malignant intraocular tumor in adults, and effective clinical treatment strategies are still lacking. Autophagy is a lysosome-dependent degradation system that can encapsulate abnormal proteins, damaged organelles. However, dysfunctional autophagy has multiple types and plays a complex role in tumorigenicity depending on many factors, such as tumor stage, microenvironment, signaling pathway activation, and application of autophagic drugs.

**Methods:**

A systematic review of the literature was conducted to analyze the role of autophagy in UM, as well as describing the development of autophagic drugs and the link between autophagy and the tumor microenvironment.

**Results:**

In this review, we summarize current research advances regarding the types of autophagy, the mechanisms of autophagy, the application of autophagy inhibitors or agonists, autophagy and the tumor microenvironment. Finally, we also discuss the relationship between autophagy and UM.

**Conclusion:**

Understanding the molecular mechanisms of how autophagy differentially affects tumor progression may help to design better therapeutic regimens to prevent and treat UM.

## Introduction

Uveal melanoma (UM) is the most common intraocular malignancy in adults, originating from melanocytes in the uvea, which includes the pigmented tissue of the iris, ciliary body, and choroid (Bande et al. 2020; Jager et al. 2020; Smit et al. 2020). The 10-year mortality rate of UM is approximately 40%. Almost half of UM patients develop metastases, mainly in the liver. Once metastases are detected, the survival time is reduced to less than 1 year (Carvajal et al. 2017; Egan et al. 1988; Singh et al. 2005). Epidemiologically, risk factors for developing UM include prolonged UV exposure; vitamin D3 deficiency (Broggi et al. 2020; Mallet et al. 2014); and presence of choroidal nevus, cutaneous dysplastic nevus syndrome, oculo-dermal melanocytosis and type 1 neurofibromatosis (Broggi et al. 2020; Krantz et al. 2017). These risk factors may be important external causes of tumorigenesis. UM frequently manifests visually as a domed or ring-shaped mass that extends into the posterior chamber of the eye. In some cases, hemorrhagic and/or necrotic foci may be observed, as well as extraocular dilatation and retinal detachment. Hyperpigmentation in UM can manifest in many degrees, from highly pigmented to grey coloured masses (Foti et al. 2021a, b). Three histological subtypes of UM have been identified by the American Joint Committee on Cancer (AJCC): (1) the spindle cell type, which is made up of spindle A and B cells and has a spindle cell morphology in 90% of tumours; (2) the epithelioid cell type, which has an epithelioid cell morphology in 90% of tumours; and (3) the mixed cell type. Other rare morphological variants have been described: (a) diffuse UM, defined as tumours involving at least one-quarter of the chylomicron; (b) clear cell UM, distinguished by a diffuse clear cell morphology caused by glycogenolysis after fixation; and (c) balloon cell UM, characterised by giant tumour cells with extensive lipid-rich cytoplasm (Barbagallo et al. 2023; McLean et al. 1983). UM treatment aims to preserve the eye and its functionality while preventing metastatic progression. Ionising radiation used in radiotherapy, a frequent conservative therapy, kills cells both directly by rupturing chemical bonds and indirectly by creating harmful free radicals. The therapeutic theory identifies two effects of radiotherapy on tumor cell viability: (1) necrosis of tumor tissue, followed by reabsorption of necrotic cellular debris by macrophages; and (2) tumor sterilisation, which is defined as the cessation of mitotic activity, interruption of growth, and reduced capacity for metastasis (Foti et al. 2021a; Zinn et al. 1981). However, radiation therapy comes with a number of ocular and periocular side effects. The genesis, development, and tumor metastasis of cancer are all influenced by hereditary chromosomal and genetic abnormalities. Numerous functional and quantitative chromosomal and gene problems in important biological pathways (such as cell cycle regulation, signalling, apoptosis, or angiogenesis) have been found and reported in UM. Tumor biology is affected by certain genetic characteristics at the level of chromosomal or gene alterations, which result in aggressive phenotypes (metastatic, hyporesponsive, and low survival). The focus of precision medicine in cancer has shifted to the identification of driver mutations for diagnostic, prognostic, and therapeutic purposes. The most frequently mutated genes regarded as UM drivers are BAP1, EIF1AX, GNA11, GNAQ, and SF3B1 (Chattopadhyay et al. 2016; Staby et al. 2018). These genes are responsible for the formation and progression of UM, respectively. Despite advances in exploring the molecular mechanisms of UM, current treatment options for this disease, such as charged particle radiotherapy, proton beam therapy, photodynamic therapy, and surgical resection, remain ineffective for overall survival (Tarlan and Kıratlı 2016). Therefore, the interaction between extrinsic and intrinsic factors in tumor formation is still the foundation for understanding the molecular pathogenesis of UM.

Autophagy is an evolutionarily conserved catabolic process involving the multistep degradation of misfolded proteins, damaged organelles, and foreign pathogenic microorganisms (Klionsky and Emr 2000; Levine and Kroemer 2008). It maintains cellular homeostasis under normal physiological or stressful conditions, which are closely associated with cellular senescence, neurodegeneration, and cancer development (Mizushima et al. 2008; Parzych and Klionsky 2014). Interestingly, autophagy is thought to play a dual role in tumorigenesis. Autophagy suppresses tumor development by removing damaged organelles and reducing oxidative stress to alleviate DNA damage or genomic instability. However, tumor cells can counteract the harsh environments of hypoxia and nutrient deficiency through activation of the autophagic pathway accompanied by further tumor maturation (White 2015). Similarly, the function of autophagy in UM is also multifaceted. For example, the long noncoding RNA ZNNT1 can inhibit the tumorigenicity of UM by promoting autophagy (Li et al. 2020a). However, overexpression of AXIIR can promote autophagy, and its combination with the autophagy inhibitor chloroquine (CQ) can enhance AXIIR-induced apoptosis (Zhang et al. 2016). As a result, the development of targeted autophagy-related drugs is considered a promising strategy for the treatment of various cancers. It is obvious that autophagy is increasingly becoming a potential target for cancer therapy. Meanwhile, autophagy is closely associated with the tumor microenvironment. It is well known that the tumor microenvironment contains not only malignant cancer cells but also many different cell types, all of which utilize or rely on autophagic processes at different times. Indeed, as will be discussed in this review, autophagy affects not only tumor cells but also cancer-associated fibroblasts (CAFs), mesenchymal stem cells (MSCs), endothelial cells, and immune cells (Folkerts et al. 2019). Therefore, it is essential to explore the function of autophagy and its tumor-targeted therapy in the context of the multiple cellular components of the tumor microenvironment. Uveal melanoma, which originates inside the eye, develops in a special immune-privileged environment that has suppressive effects on both the innate and adaptive immune systems. Currently, there is a lack of evidence that the combination of radiotherapy and immunotherapy is beneficial to the prognosis of uveal melanoma patients (Tagliaferri et al. 2022; Taylor 2016). It is difficult to treat metastatic UM. Although immune infiltrates are a hallmark of metastatic melanoma, a limited response to immune checkpoint inhibitor treatment has been observed. Recent research has described an exceptional immune response in UM patients who carry the MBD4 mutation. This new evidence suggests that certain subtypes of uveal melanoma may benefit from immunotherapy in addition to radiotherapy or chemotherapy (Rodrigues et al. 2019; Rossi et al. 2019). The goal of immunotherapy is to modulate and enhance the antitumoral immune response in patients with uveal melanoma. This finding provides potential for the future.

In this review, we first briefly define autophagy and describe the mechanisms of autophagy and the application of autophagic drugs, and then we discuss the function of autophagy on various components of the tumor microenvironment and the impact of autophagy on UM progression. Furthermore, we provide strategies for implementing autophagy-targeted drugs in cancer to counter the dual role of autophagy in tumor microenvironment components.

## Definition of autophagy

**Fig. 1:**
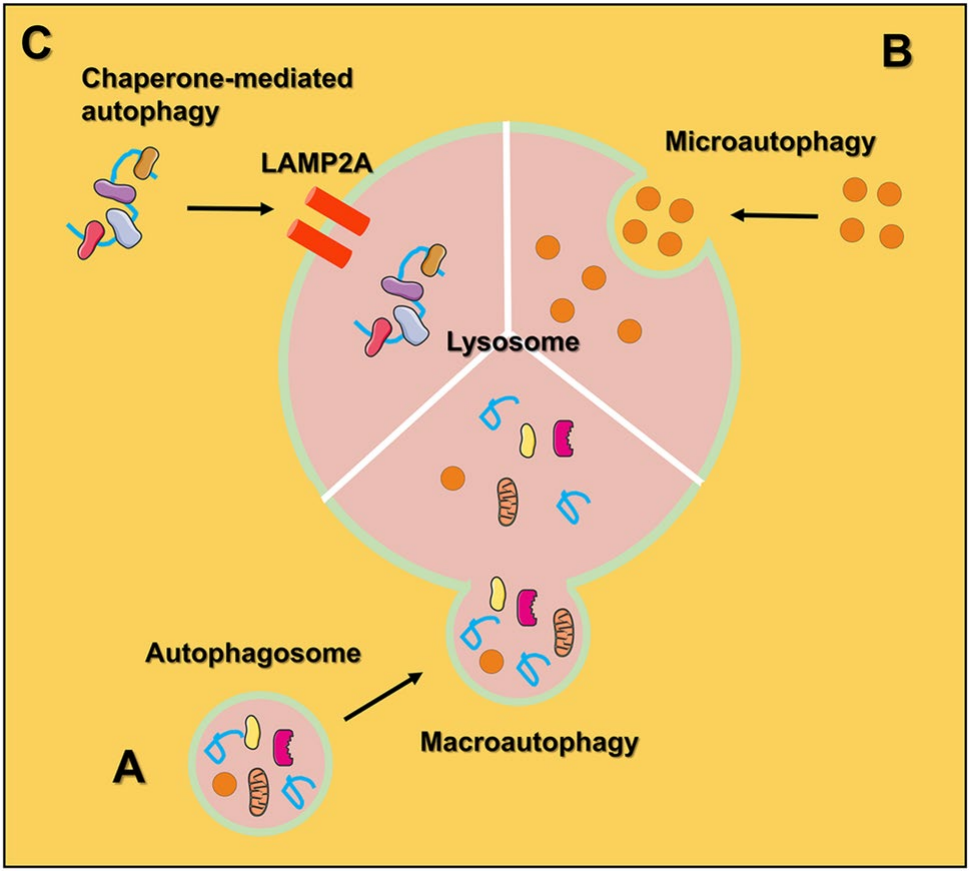
The schematic illustration of the autophagy types. **A** The process of macroautophagy formation; **B** the process of microautophagy formation; **C** the process of chaperone-mediated autophagy formation

Autophagy is a self-digestion process after the organism is stimulated by external environmental factors, in which cells degrade their own damaged, degenerated, or senescent macromolecules as well as organelles through lysosomes (Levy et al. 2017; Li et al. 2021). Autophagy is also a self-protection mechanism that is widely found in eukaryotic cells and plays an important role in the regulation of cell survival and death (Amaravadi et al. 2019; Devis-Jauregui et al. 2021). Mammalian cellular autophagy can be divided into three types according to the ways intracellular substances are transported to lysosomes and their physiological functions (Mah and Ryan 2012): (1) macroautophagy; (2) microautophagy; (3) chaperone-mediated autophagy (CMA) (Fig. 1).

(1) Autophagy frequently reported in the literature is mainly macroautophagy. In the process of macroautophagy, soluble macromolecules in the cytoplasm and denatured organelles are wrapped by single or double membranes derived from the endoplasmic reticulum or mitochondria to form autophagosomes. Then, the outer membrane of the autophagosome fuses with the lysosomal membrane to further form the autolysosome, and the materials within the autophagosome are degraded by a series of hydrolytic enzymes. Finally, the whole process of autophagy is completed (Levy et al. 2017). (2) Microautophagy is the invagination of the lysosomal membrane, which wraps and engulfs substrates in the cell and further degrades them inside the lysosome. The main difference between microautophagy and macroautophagy is that the cellular degraded component is directly wrapped by the lysosome in the process of microautophagy, and there is no process of autophagosome formation (Eskelinen and Saftig 2009). (3) In the process of CMA, degraded substrates are soluble protein molecules. Molecular chaperone proteins can recognize substrate protein molecules with specific amino acid sequences and bind to them, which are then transported to the lysosome via the receptor Lamp2a on the lysosomal membrane; the substrate protein molecules are then inside the lysosome and degraded by hydrolases. Thus, CMA, unlike macroautophagy and microautophagy, is selective in degrading proteins. In contrast, there is no significant selectivity in degrading proteins during the processes of macroautophagy and microautophagy (Mizushima et al. 2008).

## Mechanisms of autophagy

**Fig. 2:**
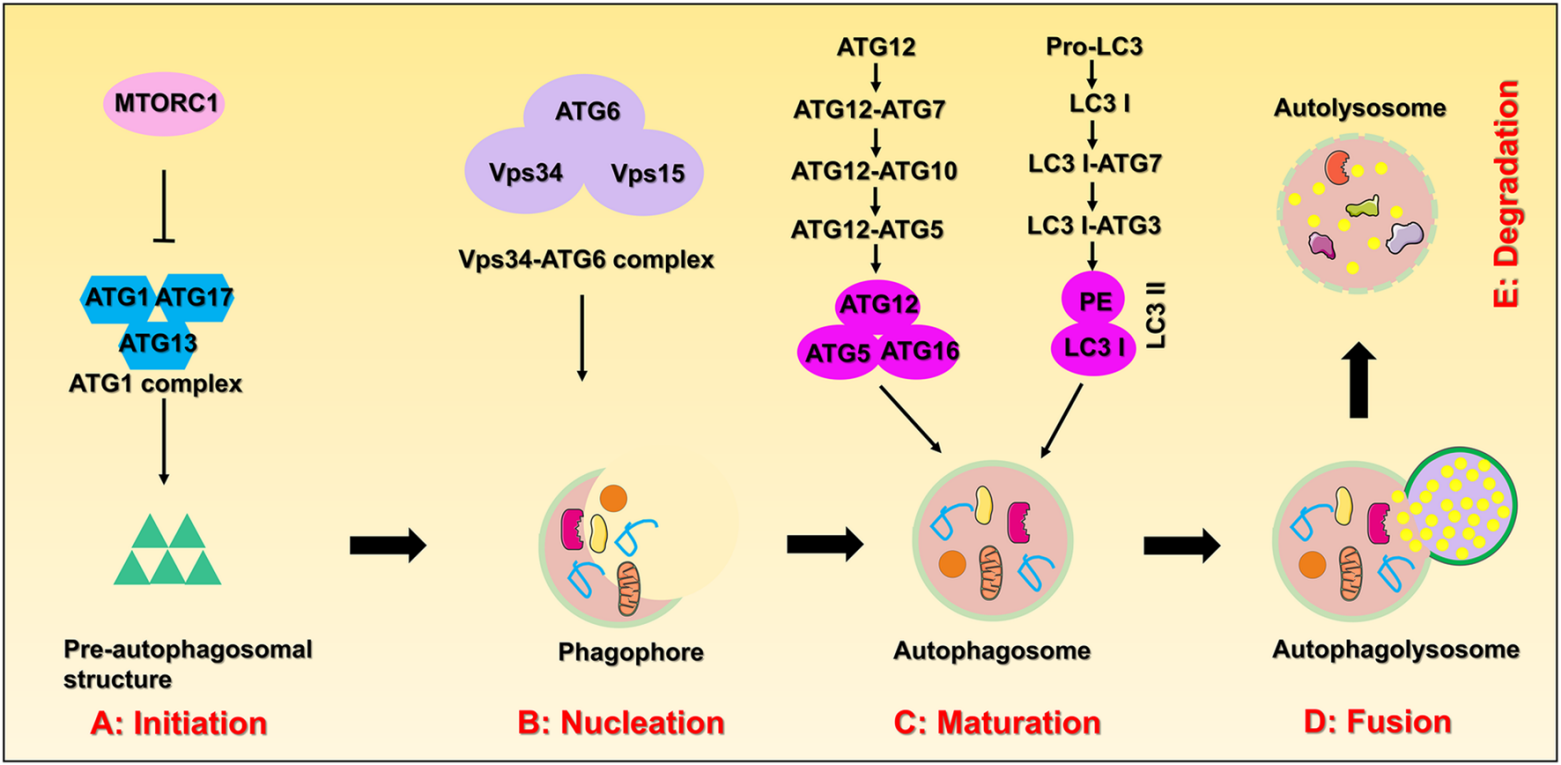
The schematic illustration of the autophagy mechanism. **A** Initiation: activation of multiple ATG proteins are engaged and localized to Pre-autophagosomal structure; **B** Nucleation: ATG proteins and lipids are recruited to form phagophore; **C** Maturation: completion and transport of the autophagosome by ATG proteins; **D** Fusion: docking and fusion between autophagosome and lysosome; **E** Degradation: degradation of the cargos inside the autolysosome.

Autophagy is a continuous process of cytological changes that can be artificially divided into five phases: (1) initiation; (2) nucleation, (3) maturation; (4) fusion; (5) degradation (Li et al. 2020b). Since the molecular mechanism of autophagy is extremely complex, we will elaborate on the mechanism of autophagy from these five aspects (Fig. 2).

### Initiation

Under normal conditions, cells can maintain low levels of basal autophagy. This is because the intracellular energy is sufficient, and the mammalian target of rapamycin protein complex 1 (mTORC1) is in the activated state. The activity of mTORC1 is inhibited when cells are under conditions of energy deficiency, abnormal protein accumulation, stress, etc. (Dan et al. 2014). The phosphorylation level of the ATG13 protein is also reduced, which leads to the rapid activation of autophagy. The initiation of autophagy in mammals is mediated by the ATG1 complex (ULK1 complex), which contains mainly ATG1, ATG13 and ATG17 proteins. The phosphorylation level of ATG13 decreases after external stimulation, resulting in the formation of a complex between dephosphorylated ATG13 and ATG1, which interacts with ATG17 to produce the ATG13-ATG1-ATG17 complex and further induces downstream autophagosome formation (Chen and Klionsky 2011; Hosokawa et al. 2009).

### Nucleation

The process of nucleation is closely related to the Vps34-ATG6 complex (PI3K-Beclin-1 complex). Here, the activated ATG1 complex regulates the activity of the Vps34-ATG6 complex, which contains the regulatory protein kinase Vps15. The Vps34-ATG6 complex also recruits ATG12-ATG5, ATG16 multimers, or LC3 protein and promotes phagocytic vesicle extension and expansion through the latter two (Feng et al. 2016; Park et al. 2016; Pattingre et al. 2005; Petherick et al. 2015).

### Maturation

The molecular mechanism of the maturation process is the most complicated and mainly depends on two ubiquitination-like systems: (a) the binding process of ATG12 and (b) the modification process of LC3. The binding of ATG12 requires the involvement of ubiquitin-activating enzymes E1 and E2. ATG12 is first activated by the E1-like enzyme ATG7, transported by the E2-like enzyme ATG10, bound to ATG5, and then bound to ATG16 to produce the ATG12-ATG5-ATG16 multimeric complex. This complex is localized on the outer membrane surface of the preautophagosomal structure and is involved in expansion of the preautophagosomal outer membrane (Mizushima et al. 2011; Shpilka et al. 2011; Tsuboyama et al. 2016). The modification process of LC3 also requires the participation of ubiquitin-activating enzymes E1 and E2. The LC3 precursor is processed into cytoplasmic soluble LC3-I by ATG4 and then covalently linked with phosphatidylethanolamine (PE) to become lipid-soluble LC3-PE (LC3-II) under the action of the E1-like enzyme ATG7 and E2-like enzyme ATG3, which is involved in membrane extension. LC3-II can bind to newly formed membranes until autolysosome formation occurs (Hanada et al. 2007; Nakatogawa et al. 2007; Tanida et al. 2004).

### Fusion

This process mainly involves the fusion of autophagosomes and lysosomes to form autolysosomes. Since autophagosomes can form randomly in the cytoplasm and lysosomes are mainly found in the perinuclear region, mature autophagosomes need to be transported through the microtubule backbone to the perinuclear region to bind with lysosomes (Kriegenburg et al. 2018; Nakamura and Yoshimori 2017). The related proteins involved in autolysosome formation include LAMP1, LAMP2, and UVRAG (Jäger et al. 2004; Tanaka et al. 2000).

### Degradation

The degradation process involves the cleavage of the autolysosome membrane and the degradation of the contents by the action of lysosomal hydrolases. The amino acids and some proteins produced during the degradation process can be used as intracellular energy for growth and differentiation (Li et al. 2020b; Panda et al. 2015).

## Application of autophagy inhibitors

Not surprisingly, a growing number of studies have demonstrated that drug resistance in cancer therapy can be eliminated by the inhibition of autophagy-associated genes or key genes in the autophagic pathway. Additionally, there is also an increasing level of interest in searching for more efficient and targeted pharmacological autophagy inhibitors (Kocaturk et al. 2019; Liu et al. 2020). We classified autophagy inhibitors into three groups according to the different molecular mechanisms of autophagy formation.

(1) Inhibitors of autophagosome formation: the Class III PI3K inhibitors 3-methyladenine (3-MA), wortmannin, LY294002, SAR405, and viridiol have been shown to prevent autophagosome formation (Pasquier 2015; Rubinsztein et al. 2012). For example, 3-MA causes radiation sensitization of esophageal cancer by inhibiting autophagy (Chen et al. 2011). Similarly, 3-MA can enhance 5-FU and cisplatin-induced cell apoptosis in colon and lung cancer (Li et al. 2009; Liu et al. 2013), respectively. Additionally, SAR405-mediated autophagy inhibition can be combined with the mTOR inhibitor everolimus to reduce the proliferation of renal tumor cells (Pasquier 2015).

(2) Lysosomal acidification inhibitors: Lysosomal promoters, including chloroquine (CQ), hydroxychloroquine (HCQ), Lys0569 and monensin, prevent lysosomal acidification and thus inhibit the degradation of material in autophagosomes (Kocaturk et al. 2019). For example, treatment with CQ combined with bevacizumab is more effective in controlling the growth of colon cancer cells (Selvakumaran et al. 2013). Likewise, the addition of CQ to glioblastoma treatment can enhance the killing effect of chemotherapy on tumor cells (Sotelo et al. 2006).

(3) Autophagosome-lysosome fusion inhibitors: Vacuolar-ATPase inhibitors, including variants of bafilomycin (BafA1, BafB1, and BafC1) and concanamycin variants (Con A, Con B, and Con C), disrupt the fusion of autophagosomes with lysosomes, while spautin-1 targets the Beclin-1 subunit of the Vps34 complex (Bowman et al. 2004; Shao et al. 2014). For example, the combination of BafA1 and 3-MA can enhance the ability of nedaplatin to kill cisplatin-resistant nasopharyngeal carcinoma cells (Liu et al. 2015). Additionally, according to the literature, BafA1 can increase chemosensitivity in gastric cancer, osteosarcoma and colon cancer cells (Greene et al. 2013; Li et al. 2016; Xie et al. 2014).

## Application of autophagy inducers

Excessive activation of autophagy is a mechanism that accelerates cell death, and induction of autophagy may be a strategy to promote tumor cell death. It has been reported in the literature that certain tumor cells can resist the apoptotic pathway, which allows them to escape death (Kocaturk et al. 2019; Yang et al. 2013). Thus, autophagy plays an important role as an alternative cell death pathway in tumor cells with apoptosis resistance. Several articles have been published that discuss the possible therapeutic effects of autophagy activation on a range of diseases. Currently, the exploration of autophagy activators related to autophagy has become a research hotspot due to their potential clinical value. Here, we will briefly discuss some of the chemical agents known to act directly on the autophagic pathways. The mTOR signaling pathway plays an important inhibitory role in autophagy formation, and mTOR is considered a promising drug target for cancer therapeutic strategies (Cheong et al. 2012). The mTOR inhibitor rapamycin has been reported to enhance the sensitivity of tumor cells to radiation therapy and even directly inhibit cell proliferation in malignant glioma (Takeuchi et al. 2005). Resveratrol, a natural plant derivative, is an important anticancer compound that activates PI3K-AKT, WNT/β-Catenin and other signaling pathways to induce autophagy, thereby promoting tumor cell death (Fu et al. 2014; Jiang et al. 2009; Wang and Feng 2015). An increasing number of studies have shown that the anticancer effects of ginsenosides are attributed to the induction of the autophagic pathway. For example, ginsenoside F2 promotes autophagy in breast cancer stem cells (Mai et al. 2012). Similarly, ginsenoside Rb1 promotes autophagy in colon cancer cells through the generation of reactive oxygen species and activation of the JNK signaling pathway (Kim 2013). Lithium induces autophagy along with autophagosomes and autophagic lysosomes in several cancers, including melanoma, hepatocellular carcinoma, cervical carcinoma, and renal carcinoma (Yang et al. 2023). Lithium agents such as lithium acetoacetate (LiAcAc), lithium chloride (LiCl), lithium citrate (Li_3_C_6_H_5_O_7_) and lithium carbonate (Li_2_CO_3_) can be used to induce autophagy and thus inhibit tumor growth (Villegas-Vázquez et al. 2023). There are also some small-molecule drugs that can induce autophagy to inhibit tumor growth. However, currently, these drugs are still far from entering the clinic, mainly because the autophagy mechanism is too complex, and it is difficult for one type of drug to achieve the desired therapeutic effect. This may also suggest that we need to combine multiple drugs to translate basic research on autophagy into clinical treatment.

## Autophagy and the tumor microenvironment

**Fig. 3:**
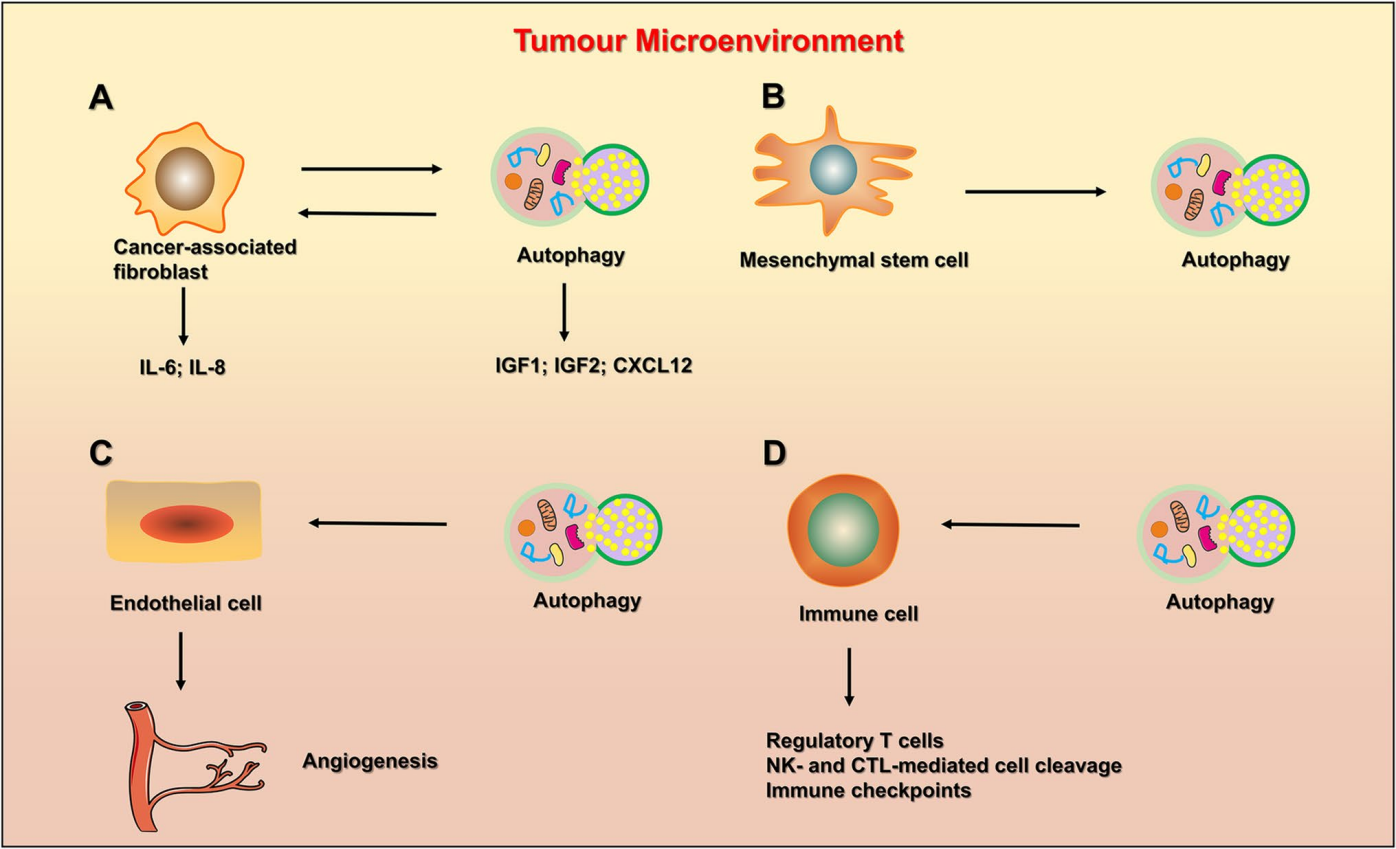
The schematic illustration of the autophagy and the tumor microenvironment. **A** Autophagy interacts with Cancer-associated fibroblast; **B** autophagy interacts with Mesenchymal stem cell; C autophagy plays a role in angiogenesis by affecting endothelial cells. **D** Autophagy can affect the regulatory T cells, NK-and CTL-mediated cell cleavage and immune checkpoints

The tumor microenvironment is a specific niche during tumor progression that plays important roles in tumor growth, survival, and response to drug therapy. The microenvironment consists of many different cell types, including cancer-associated fibroblasts (CAFs), mesenchymal stem cells (MSCs), endothelial cells, and immune cells. All these cell types promote or inhibit tumor growth through autophagy at different stages. For example, autophagy in fibroblasts promotes tumorigenesis, whereas autophagy in some immune cells promotes an antitumor immune response (Folkerts et al. 2019). Here, we will describe the effects of activation or inhibition of autophagy on several key cells in the tumor microenvironment, including CAFs, MSCs, endothelial cells, immune cells, and others (Fig. 3).

### Effect of autophagy on CAFs, MSCs, and endothelial cells in the tumor microenvironment

CAFs with activated autophagy can secrete chemokines and inflammatory factors to promote tumor growth or evade killing by chemotherapeutic agents. For example, CAFs secrete soluble factors through autophagy, including IGF1, IGF2, and CXCL12, all of which promote the survival of skin melanoma and lung cancer cells after radiotherapy (Wang et al. 2017). In addition, injection of CAFs at the eradicated tumor site accelerate tumor recurrence, which is eliminated by IGF2 knockdown or 3-MA treatment (Wang et al. 2017). Similarly, inflammatory factors secreted by CAFs, including IL-6 and IL-8, can knock down the expression of Beclin-1 protein, which attenuates the migratory ability of head and neck squamous cell carcinoma cells (New et al. 2017). At the same time, factors secreted by tumor cells can also activate autophagy in CAFs, which in turn promotes more cytokine secretion by CAFs and weakens the chemotherapy or radiotherapy sensitivity of tumor cells (Wang et al. 2017). Therefore, we should consider that inhibition of autophagy in both CAFs and tumor cells may be superior to inhibition of autophagy in tumor cells only when developing drugs for autophagy. MSCs also promote tumorigenicity through the induction of autophagy. For example, it has been reported in the literature that AML cells derived from primary patients have better proliferation capacity and survival time when cocultured with MSCs (Schuringa and Schepers 2009; van Gosliga et al. 2007). Indeed, simultaneous knockout of the ATG7 gene in MSCs and AML cells enhances the effectiveness of treatment with cytarabine compared to knockout of ATG7 in AML cells only (Piya et al. 2016). Similarly, MSCs can inhibit the apoptotic pathway in lung cancer cells by activating autophagy in the presence of nutrient deficiencies (Zhang et al. 2013). Endothelial cells are one of the most important cells in the composition of blood vessels. Tumor growth cannot occur without oxygen and nutrients transported by neovascularization. This shows that inhibition of endothelial cell growth is very effective in the treatment of tumors.

### Effect of autophagy on immune cells in the tumor microenvironment

Immune cells are one of the most important components of the tumor microenvironment, and their immune function is also influenced by autophagy. For instance, T-cell immunity is a multilayered and intricate regulatory process, and autophagy may play different roles at various stages. Autophagy in cancer cells suppresses anticancer immunity by decreasing sensitivity to NK and CTL-mediated lysis. Autophagy in cancer cells can regulate the expression of immune checkpoints (Folkerts et al. 2019). Therefore, we will discuss the role of autophagy in immune cells from these three aspects.

Regulatory T (Treg) cells are a subpopulation of CD4 + T cells that are strongly associated with survival in tumor patients. It has been reported in the literature that knockout of autophagy genes induced Treg cell apoptosis and blocked Treg-mediated suppression of effector T-cell responses (de Jong et al. 2009; Zhou et al. 2017). Similarly, treatment with the autophagy inducer rapamycin enhanced specific CD8 + T-cell responses in aged mice but not in ATG7 knockout aged mice (Phadwal et al. 2012; Puleston et al. 2014). In addition, the proliferation and survival of T cells are dependent on an active autophagic pathway. Therefore, systemic application of autophagy inhibitors may suppress anticancer immune responses. In fact, in vitro treatment of T cells with the autophagy inhibitor CQ reduced T-cell-dependent cell cleavage, depressed the ability of T cells to proliferate and reduced cytokine secretion (Schmidt et al. 2017).

Several studies have illustrated that activation of autophagy in cancer cells can reduce the efficacy of NK- and CTL-mediated cell cleavage. For example, autophagy in cancer cells can affect the stability of immune synapses generated between immune cells and their target cells. In particular, the formation of gap junctions requires connexins (e.g., connexin-43), which normally promote the exchange of small molecules between effector and target cells and are necessary for the cleavage of NK cells (Tittarelli et al. 2014). Importantly, the gap junction protein connexin-43 can translocate active granzyme B (one of the major cytotoxic molecules of CTLs and NK cells) into target cells (Tittarelli et al. 2014). In conclusion, activation of autophagy in cancer cells negatively affects NK- and CTL-mediated cell cleavage through degradation of granzyme B and inhibition of immune synapses.

Immune checkpoints are coinhibitory receptor/ligand pairs used to inhibit immune cell activity (Pardoll 2012). One prominent example is the receptor programmed cell death 1 (PD-1) and its ligand (PD-L1), which is a key inhibitor of anticancer T-cell responses in the tumor microenvironment (Freeman et al. 2000). Interestingly, activation of autophagy using the mTOR inhibitor rapamycin reduced PD-L1 expression in lung cancer cells in vitro and in vivo, while activation of mTOR increased PD-L1 expression (Lastwika et al. 2016). Similarly, melanoma and ovarian cancer cells with low levels of autophagy expressed higher levels of PD-L1 than cells with high levels of autophagy (Clark et al. 2016). In conclusion, inhibition of the autophagic signaling pathway was associated with increased PD-L1 expression.

## Studies on autophagy and UM

As mentioned above, autophagy plays an important role in tumor formation, and it is currently challenging to develop autophagy-related targeted drugs. Therefore, we need to learn more about the function of autophagy in other diseases, which will help us to develop different therapeutic strategies for autophagy in different diseases. Here, we will briefly introduce research on autophagy in UM from three aspects, including basic research on autophagy, autophagy genes and the prognosis of tumor patients and the exploration of drug targets for autophagy.

### Basic research on autophagy in UM

Hypoxia is a common characteristic of malignant tissues, owing to an inadequate, and sometimes immature, vascular network that precludes appropriate blood and oxygen perfusion. According to the findings of several scientific experiments, hypoxia is a powerful stress stimulus that can induce autophagy in both healthy and cancerous cells (Giatromanolaki et al. 2004; Sivridis et al. 2003). Under low oxygen tension, the hypoxia-inducible factor HIF1A, which is a key transcription factor, is related to the regulation of autophagy in neoplastic cells. HIF1A is also linked to the regulation of the expression of a variety of genes that participate in angiogenesis and anaerobic metabolism (Rouschop and Wouters 2009; Semenza et al. 1996; Zhang et al. 2008). BECN1 (sometimes referred to as Beclin 1) is the mammalian homolog of the yeast gene Atg6, which is required for the development of autophagosomes. The human Beclin 1 gene is located on chromosome 17q21, and its copy in the majority of breast, ovarian, and prostate cancer cell lines is missing due to a deletion of a single copy. In addition, mice that lack the Beclin gene are more likely to develop tumors of their own accord, which provides credibility for the theory that BECN1 possesses a tumor suppressor function and plays a role in the regulation of autophagy (Aita et al. 1999; Karantza-Wadsworth and White 2007; Qu et al. 2003). It has been determined that autophagy is increased in more than 40–50% of uveal melanomas and that these cases are connected with tumor hypoxia. Because of its interaction with hypoxia and acidity in UM, overexpression of BECN1 was found to be associated with early metastases and poor prognosis. Conversely, there is a strong association between underexpression of BECN1 protein and late metastases/better prognosis (Giatromanolaki et al. 2011). However, contrary conclusions have been drawn regarding the contribution of Beclin 1 to autophagy in UM. There is evidence that Beclin-1 has a positive prognostic effect in uveal melanoma, with higher immunohistochemistry levels of the protein being associated with better outcomes in terms of metastasis risk and overall survival (Broggi et al. 2020). In various types of human cancer, including melanoma of the skin, autophagy-associated cell death has emerged as a significant immunogenic pathway that can enhance the tumor response to treatment. Autophagy has been demonstrated to play crucial roles in dendritic cell and T-lymphocyte infiltration in immune-competent animal models, and current research suggests that targeting autophagy in melanoma cells through combination with immunotherapy might improve effects in boosting tumor regression (Bustos et al. 2018; Nicotra et al. 2010; Sun et al. 2018). These findings are intriguing because they suggest that treating UM with autophagy-targeting therapies may provide a fresh opportunity for individuals who have not responded to targeted and immunotherapeutic procedures now active against the vast majority of human solid malignancies. This shows that more evidence is needed to assess the prognostic value of Beclin-1 for UM patients, as well as targeted autophagic modulation for the treatment of UM. Contradictory and complex interactions exist between apoptosis and autophagy. Autophagy may function as an upstream signal of apoptosis when these two processes work together to induce cell death. In contrast, autophagy may counteract apoptotic cell death under certain conditions (Amaravadi et al. 2007). It has been shown that overexpression of Annexin A2 receptor (AXIIR) can induce apoptosis by activating caspase-3, caspase-8, and caspase-9. Moreover, overexpression of AXIIR can promote autophagy, and its combination with the autophagy inhibitor CQ can enhance AXIIR-induced apoptosis (Zhang et al. 2016). This suggests that AXIIR overexpression-induced autophagy prevents apoptosis in UM cells. However, a few studies have shown that activation of autophagy can inhibit the progression of UM. For example, the long noncoding RNA ZNNT1 can inhibit the tumorigenicity of UM by promoting autophagy and can also regulate the expression of key autophagy-related proteins. Overexpression of ZNNT1 increased the expression of ATG12, which reduces the potential of UM to metastasize and cause tumors while promoting cellular autophagy. In vivo tests demonstrated that ZNNT1 could stop melanoma from growing in naked mice, and that ZNNT1's tumour suppressor effect could be somewhat mitigated by knocking down ATG12 (Li et al. 2020a). PTK6, a member of the tyrosine kinase family, inhibits autophagy through the mTOR pathway, which further promotes the progression of UM. PTK6 is also highly expressed in UM samples and correlates with the prognosis of tumour patients, and experiments in nude mice have demonstrated that PTK6 promotes the growth of UM (Liu et al. 2023b). LINC01278 is lowly expressed in UM, and the tumour patients with high expression of LINC01278 have a better prognosis. Meanwhile, LINC01278 inhibited the development of UM by activating autophagy, which was also demonstrated by in vitro experiments (Liu et al. 2023a). D-type cyclins (CCNDs), among the most often dysregulated therapeutic targets in human cancer, play a crucial role in regulating the cell division cycle. AMBRA1 regulates UVM cell proliferation by promoting CCND1 ubiquitination and degradation. Accordingly, research supports the idea that AMBRA1 is a crucial tumor suppressor that inhibits the proliferation of UVM cells (Zhao et al. 2022). The BRAF mutation increases the risk that melanoma may progress to a terminal stage. Many different kinds of cancer are affected by autophagy triggered by BRAF mutation inhibitors. Inhibition of BRAF increases autophagy in UM, which is a result of the endoplasmic reticulum (ER) stress response. Autophagy may be partly reversed by inhibiting the ER stress response, which might also reduce the antitumor impact of BRAF inhibition in UM (Zhao et al. 2017). Taken together, autophagy may play a dual role in the progression of UM. More research is needed to explore the role of autophagy in UM.

### Autophagy-related genes and prognosis of UM patients

Currently, with the application of second-generation sequencing in clinical areas, there is an increasing number of studies on transcriptome sequencing analysis of UM patients. For example, the tumor immune microenvironment (TIME) is also strongly associated with angiogenesis, which is a key process in the formation and progression of UM. CARD11, which is mostly expressed in lymphoid tissues, has been shown to be connected with immune cells and is known to have a significant role in the process of carcinogenesis (Shi et al. 2021). This study not only suggested that the expression of CARD11 and immune cell infiltration play important roles in the development of UM but also revealed a viable research direction for immunotherapy. Zheng et al. uncovered nine prognostic autophagy-related genes and found that high expression levels of IKBKE, BNIP1, ITGA6, and FKBP1A and low expression levels of DLC1, PRKCD, GABARAPL1, LMCD1 and TUSC1 were associated with worse prognosis of UM (Zheng et al. 2021). In addition, there was an increase in immune cell infiltration and an enrichment of tumor hallmarks in UM patients with higher risk scores. Meanwhile, Chuah et al. added some data supporting that these nine autophagy-related genes were significantly associated with the enrichment of cancer features, including angiogenesis, IL6–KJAK–STAT3 signaling, reactive oxygen species pathways and oxidative phosphorylation (Chuah and Chew 2021). A lysosomal catabolic process called autophagy is modulated by noncoding RNAs such as miRNAs and lncRNAs. According to both experimental and clinical research, autophagy-related lncRNAs have been shown to have important diagnostic and therapeutic benefits in a variety of malignancies, including UM. For example, autophagy-associated long noncoding RNAs can be used as biomarkers of clinical prognosis in UM patients (Chen et al. 2021; Cui et al. 2021). The literature shows that SOS1-IT1, AC016747.1, AC100791.3, and AC018904.1 are risk indicators, and the prognosis of UM patients with high expression of these four genes is poor. In contrast, AC104825.1 and AC090617.5 were protective factors, and UM patients with high expression of these two genes had a better prognosis. This signature could assist in the identification of high-risk UM patients and help researchers to elucidate the molecular mechanism of autophagy-related lncRNAs in UM pathogenesis. However, these studies are only bioinformatic analyses, and more experiments are needed for verification.

### Exploration of autophagic drug targets in UM

UM is the most common primary intraocular malignancy in adults. Therapeutics that have been shown to be beneficial in treating cutaneous melanoma have minimal effectiveness in treating UM, which may be attributable to the fact that UM has a low mutational load. For this reason, novel pharmacological therapies are desperately needed for UM. Autophagy has been reported to play an important role in the progression of UM, so it is a viable strategy for exploiting autophagic drugs for UM treatment. Elaiophylin is a late-stage autophagy inhibitor that showed remarkable anticancer efficacy in human UM cell lines. This effect was achieved by inhibiting mitophagy, increasing oxidative stress, and ultimately causing autophagic cell death (Zhu et al. 2022). Over 90% of uveal melanomas harbor mutations in GNAQ or GNA11 (GNAQ/11), which activate carcinogenic pathways such MAP kinase and YAP. Autophagy was induced by blocking GNAQ/11 stimulation of MAP kinase signaling. This study provides more evidence that YAP, MEK1/2, and lysosome function are essential therapeutic targets for GNAQ/11-driven melanoma and finds that the combination of trametinib and hydroxychloroquine was an effective treatment for metastatic uveal melanoma (Ambrosini et al. 2013; Truong et al. 2020). Cuprous oxide nanoparticles (Cu2O-NPs) can impede the proliferation of cancer cells and impair the capacity of UM cells to migrate and invade. One possible mechanism is that Cu2O-NPs cause damage to mitochondria, autophagolysosomes, and lysosomes, which then increases the amount of reactive oxygen species and overstimulates apoptosis and autophagy (Song et al. 2015). The findings provide useful background information on Cu2O-NPs for future applications and demonstrate that Cu2O-NPs have therapeutic potential for uveal melanoma. Research on the exploitation of autophagic drugs applied to UM therapy is still insufficient. Therefore, we need to strengthen the research on autophagy in UM and expect to develop specific autophagy-related drugs for UM patients.

### Investigation of autophagy in other tumor types

The autophagy protein p62/SQSTM 1/Sequestosome-1 is essential for cell metabolism, proliferation, and malignant development. A pilot study that included 45% of primary and 55% of recurrent glioblastoma (GBM) cases with isocitrate dehydrogenase (IDH) 1/2 wild-type showed immunoexpression of p62 in the nucleus and cytoplasm of tumor components (Ieni et al. 2022). Therefore, the autophagy-associated protein p62 can be a focus of targeted therapeutics. According to an early report, the autophagy protein ATG7 may be a good predictor of prognosis for malignant pleural mesothelioma (MPM) (Rapisarda et al. 2021). Preliminary research on melanoma revealed a negative correlation between worse treatment response and increased autophagy. Tumour growth suppression and a considerable extension of overall survival were demonstrated using a model of autophagy inhibition by ATG7 deletion in melanoma driven by Braf activation and Pten allele loss (Ma et al. 2011; Xie et al. 2015). A study using xenografts of breast cancer cells has also demonstrated the function of autophagy in the control of metastasis. Two crucial regulators of the epithelial-mesenchymal transition (EMT) and metastasis are twisted family bHLH transcription factor I (TWISTl) and snail family transcriptional repressor I (Snail), which are both stabilised by VPS34 and degraded by autophagy in non-metastatic cells by DNA-binding protein (DEDD) (Lv et al. 2012). The role of autophagy has also been explored in the p53^−/−^ lung cancer model, which showed more aggressive adenocarcinomas than those in p53 wild-type mice. Dual ablation of autophagy and p53 led to an increase in tumor volume, suggesting a role for p53 in inhibiting tumorigenesis following autophagy (Guo et al. 2013; Rao et al. 2014). Pancreatic cancer is associated with increased autophagy, and autophagy has been linked to tumor growth by ATG5 knockdown suppression of autophagy. In Kras-driven tumors, ATG5 was eliminated entirely or reduced to a single allele in order to control autophagy. Although the ATG5 allele deletion decreased the production of tumors when compared to Kras controls, the deletion of a single ATG5 allele increased the creation and spread of tumors (Görgülü et al. 2019; Yang et al. 2011). Although the role of autophagy in GBM, MPM, melanoma, breast, lung, and pancreatic cancers has been investigated in detail, autophagy in UM has been less well studied and most of the studies have been on the inhibitory effect of autophagy in UM, and its promotional as well as dual roles have yet to be thoroughly investigated.

## Conclusions

Autophagy plays a dual role in tumorigenesis and therapy, depending on the type of autophagy, the stage of the tumor, the tumor microenvironment, etc. Autophagy has been shown to have dynamic functions in cancer, acting both as a tumor suppressor early in the evolution of the disease and as a cancer promoter later in regard to tumor persistence and therapeutic resistance. Autophagy functions as a survival pathway and quality-control mechanism in the early stages of tumorigenesis, removing damaged proteins and organelles and preventing tumor initiation while also contributing to normal cell physiology metabolism and providing biological materials and energy in response to stress. As tumors develop into their late stages, they become established and susceptible to environmental challenges such as hypoxia, nutritional deficiency, and restricted angiogenesis. Autophagy is a continual degradation and recycling system that probably contributes to the survival and growth of established tumors. It also promotes the aggressiveness of cancer by facilitating metastasis, which is another way that cancer becomes more dangerous (Ajoolabady et al. 2020; Li et al. 2020b). On the one hand, autophagy, which is a form of programmed cell death, can be found in virtually every type of cancer, serves as a mechanism for tumor suppression, helps promote the destruction of oncogenic chemicals, and ultimately prevents the formation of malignancies. The conclusion is that cancer can be caused by defective autophagy or insufficient autophagy. According to research, all chemotherapeutic drugs and radiotherapies were found to cause metabolic stress in cancer cells and a simultaneous suppression of autophagy. This suggests that controlling autophagy is a promising area on which to focus when creating new cancer treatments. On the other hand, autophagy plays a role in a number of signaling pathways during the process of tumorigenesis through its coordination with apoptosis. Autophagy promotes tumor cell survival in hypoxic or low-nutrient environments, while apoptosis suppresses cancer cell survival in these same situations, demonstrating that these two catabolic processes are crucial to both organismal homeostasis and the tumor microenvironment. The duality of autophagy is ultimately reflected in the promotion of tumor cell survival or tumor cell death, suggesting that autophagy inducers and autophagy inhibitors can be applied in tumor therapy, as illustrated by previous literature. And with regard to autophagy in UM, the following studies have been performed. BECN1, as the mammalian counterpart of the yeast gene Atg6, is necessary for the formation of autophagosomes. Over 40–50% of uveal melanomas have been found to have elevated autophagy, and these cases are linked to tumour hypoxia. Because BECN1 interacts with hypoxia and acidity in the UM, overexpression is linked to early metastasis and a poor prognosis. There is also evidence that Beclin-1 has a positive prognostic role in uveal melanoma, with higher levels of immunohistochemistry associated with a low risk of metastasis and better overall survival. Therefore, the development of targeted small-molecule drugs or drugs that selectively modulate cellular autophagy might be a reasonable and safe clinical strategy to inhibit tumor growth. This is also necessary to translate autophagy research into the clinic. A large number of mechanistic studies have demonstrated that promotion of autophagy can play a role in suppressing UM. In summary, autophagy-related genes, lncRNAs and miRNAs can inhibit the tumorigenicity of UM by promoting autophagy. However, the pro-tumorigenic effect of autophagy on UM and the dual effect on UM need to be studied in depth.

## Data Availability

Not applicable.

## Abbreviations

UM: Uveal melanoma
GNAQ: G protein subunit alpha q
GNA11: G protein subunit alpha 11
AXIIR: Annexin A2 receptor
CQ: Chloroquine
CAFs: Cancer-associated fibroblasts
MSCs: Mesenchymal stem cells
HCQ: Hydroxychloroquine
mTOR: Mechanistic target of rapamycin kinase
LC3: Microtubule associated protein 1 light chain 3 alpha
3-MA: 3-Methyladenine
PI3K: Phosphatidylinositol 3-kinase
ATG: Autophagy related
IL: Interleukin
PD-1: Programmed cell death 1
CMA: Chaperone-mediated autophagy
mTORC1: Rapamycin protein complex 1
BAP1: BRCA1 associated protein 1
EIF1AX: Eukaryotic translation initiation factor 1A X-linked
SF3B1: Splicing factor 3b subunit 1
ULK1: Unc-51 like autophagy activating kinase 1
VPS34: Vacuolar protein sorting 34
LAMP: Lysosomal associated membrane protein
UVRAG: UV radiation resistance associated
AML: Acute myeloid leukemia
IGF: Insulin like growth factor
CXCL12: C-X-C motif chemokine ligand 12
